# Antioxidant Peptides From Protein Hydrolysate of Marine Red Algae *Eucheuma cottonii*: Preparation, Identification, and Cytoprotective Mechanisms on H_2_O_2_ Oxidative Damaged HUVECs

**DOI:** 10.3389/fmicb.2022.791248

**Published:** 2022-04-21

**Authors:** Kun-Lai Sun, Min Gao, Yue-Zhen Wang, Xue-Rong Li, Peng Wang, Bin Wang

**Affiliations:** ^1^Zhejiang Provincial Engineering Technology Research Center of Marine Biomedical Products, School of Food and Pharmacy, Zhejiang Ocean University, Zhoushan, China; ^2^College of Food Science and Engineering, Ocean University of China, Qingdao, China

**Keywords:** *Eucheuma cottonii*, antioxidant peptide, cytoprotection, antioxidant enzyme, cell apoptosis

## Abstract

To screen, prepare, identify, and evaluate the activities of natural antioxidants for treating chronic diseases caused by oxidative stress. Two algal proteins, namely ZD10 and ZD60, precipitated with 10 and 60% (NH_4_)_2_SO_4_ were extracted from red algae *Eucheuma cottonii* (*E. cottonii*) and hydrolyzed using five proteolytic enzymes. The results showed that ZD60 played the most significant role in the enhancement of 2,2-diphenyl-1-picrylhydrazyl radical (DPPH⋅) scavenging activity (25.91 ± 0.24%) among all protein hydrolysates. Subsequently, six antioxidant peptides (EP1-EP6) were isolated from the papain hydrolysate of ZD60 by ultrafiltration and chromatography methods. Their amino acid sequences were identified as Thr-Ala (EP1), Met-Asn (EP2), Tyr-Ser-Lys-Thr (EP3), Tyr-Ala-Val-Thr (EP4), Tyr-Leu-Leu (EP5), and Phe-Tyr-Lys-Ala (EP6) with molecular weights of 190.21, 263.33, 497.55, 452.51, 407.51, and 527.62 Da, respectively. Of which, EP3, EP4, EP5, and EP6 showed strong scavenging activities on DPPH⋅, hydroxyl radical (HO⋅), and superoxide anion radical (O- 2⋅). Moreover, EP4 and EP5 could significantly protect human umbilical vein endothelial cells (HUVECs) from H_2_O_2_-induced oxidative damage by increasing the levels of antioxidant enzyme systems including superoxide dismutase (SOD) and glutathione peroxidase (GSH-Px) to reduce the levels of reactive oxygen species (ROS) (60.51 and 51.74% of model group) and malondialdehyde (MDA) (75.36 and 64.45% of model group). In addition, EP4 and EP5 could effectively inhibit H_2_O_2_-induced apoptosis by preventing HUVECs from early apoptosis to late apoptosis. These results indicated that the antioxidant peptides derived from *E. cottonii*, especially EP4 and EP5, could serve as the natural antioxidants applied in pharmaceutical products to treat chronic cardiovascular diseases caused by oxidative damage, such as coronary heart disease, atherosclerosis, etc.

## Graphical Abstract

The mechanisms of antioxidant peptides EP4 and EP5 on protecting HUVECs from oxidative damage.

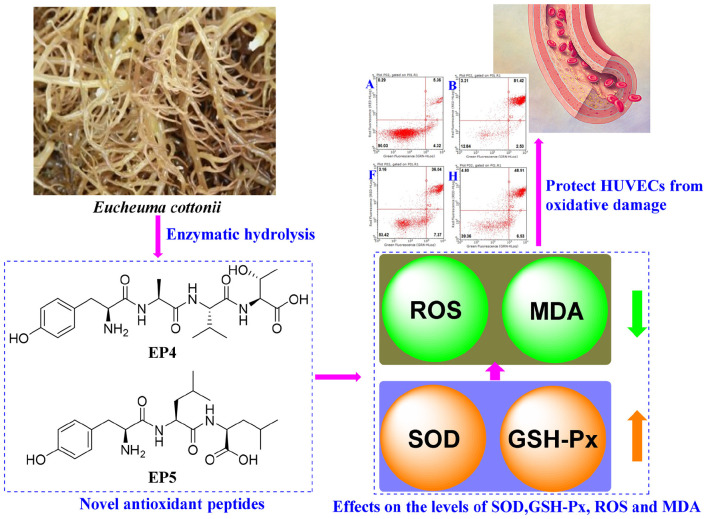

## Introduction

Reactive oxygen species are the natural byproducts of oxygen metabolism in cells and are efficiently scavenged by antioxidant defense systems including antioxidant enzymes and antioxidants under normal conditions *in vivo* (Du et al., 2016; Hou et al., 2019; Wong et al., 2020). However, the physiological equilibrium between ROS generation and elimination will be broken when the organism is under pathological conditions (including excessive pressure, smoking, ultraviolet radiation, environmental pollution, etc.) for a long time. Excessive ROS can destroy functional molecules, which further trigger many chronic diseases in the body (Harnedy et al., 2017; Rahman et al., 2018; Chen et al., 2020). Antioxidants can transform ROS into more stable forms or molecular structures to prevent the propagation of the ROS-mediated peroxidizing chain reaction by serving as a proton donor, hydrogen donor, and/or lipid peroxyl radical trap (Zhao et al., 2018; Yang et al., 2019). Furthermore, antioxidants can regulate *in vivo* antioxidant enzyme systems including GSH-Px, catalase (CAT), and SOD to reduce the damage of oxidative stress (Rahman et al., 2018; Zhang et al., 2019a; Cho and Lee, 2020). Till now, synthetic antioxidants, such as butylated hydroxyanisole (BHA), butylated hydroxytoluene (BHT), ethoxyquin, and n-propyl gallate, have been widely used in the treatment of oxidative damage in biological systems. Unfortunately, the long-term use and high doses of synthetic antioxidants in food are strictly limited due to their potential risks related to health (Botterweck et al., 2000; Peng et al., 2009). Therefore, there is an increasing interest in screening original antioxidants with low toxicity from natural sources for treating those chronic diseases (Migliore and Coppede, 2008; Reuter et al., 2010; Liu et al., 2018).

At present, some peptides with excellent antioxidant activities have been isolated from protein hydrolysates of different marine sources, such as stone fish (*Actinopyga lecanora*) flesh (Bordbar et al., 2018), skate (*Raja porosa*) cartilage (Pan et al., 2016), croceine croaker (*Pseudosciaena crocea*) muscle (Chi et al., 2015a), monkfish (*Lophius litulon*) muscle (Hu et al., 2020), mackerel (*Scomber japonicus*) muscle (Bashir et al., 2020), bluefin leatherjacket (*Navodon septentrionalis*) head (Chi et al., 2015b), cooked eggs (Wang et al., 2018), ark shell (*Scapharca subcrenata*) (Jin et al., 2018), pearl oyster (*Pinctada fucata*) muscle (Wu et al., 2017), and tuna scales (Qiu et al., 2019). Marine algae are the important protein resources, and the protein contents in brown and red algae are 5–15% and 10–47% on a dry weight basis, respectively (Ibanez and Cifuentes, 2013). Moreover, protein hydrolysates and peptides derived from marine algae showed various biological activities, such as antiatherosclerotic, antihypertensive, anticancer, and immunomodulatory activities (Fan et al., 2014). Enzymatic hydrolysates from a benthic diatom (*Navicula incerta*) exhibited significant radical scavenging effects on 2,2-diphenyl-1-picrylhydrazyl radical (DPPH⋅), HO⋅, and O- 2⋅ (Kang et al., 2011). Sheih et al. (2009) reported that eleven peptides (VECYGPNRPQF) from pepsin hydrolysate of *Chlorella vulgaris* waste showed potent antioxidant activities against different oxidation systems. Dodeca peptide (LGLDVWEHAYYL) from the catalytic center of the C-terminal SOD domain of *Arthrospira platensis* showed remarkable radical scavenging activity and could significantly reduce the intracellular ROS level in H_2_O_2_-exposed leucocytes. Moreover, LGLDVWEHAYYL did not exhibit any cytotoxic activity against the leucocytes (Sannasimuthu et al., 2018). In summary, antioxidant peptides from marine alga have shown significant bioactivities and could be applied in functional foods, even developed as biopharmaceutical antioxidant drugs with low toxicity.

*Eucheuma cottonii* is an edible tropical red macroalga and is widely cultivated throughout Southeast Asia, India, Brazil, China, and Mexico (Abu Bakar et al., 2015). The previous studies reported that the contents of proteins, dietary fibers, and carbohydrates of *E. cottonii* were 9.76 ± 1.33%, 26.49 ± 3.01%, and 25.05 ± 0.99% on a dry weight basis, respectively (Matanjun et al., 2008). κ-Carrageenan with high yield and purity from *E. cottonii* has been acted as the plasticizer together with sorbitol to produce biodegradable packaging films with good physical properties (Ili Balqis et al., 2017). The alcohol extract of *E. cottonii* could reduce oxidative stress and signaling for mucin synthesis, which was caused by chronic coal dust exposure (Kania et al., 2013). In addition, methanol extract of *E. cottonii* could dose-dependently inhibit tumor development *via* apoptosis induction and restrain erythrocyte lipid peroxidation by improving antioxidative status in the cancer-induced rats (Namvar et al., 2012). However, as far as we know, there is scarce research on the antioxidant peptides of *E. cottonii*. Therefore, the objectives of the study were to (i) isolate and identify antioxidant peptides from protein hydrolysate of *E. cottonii* and (ii) evaluate the radical scavenging activities and cytoprotective effects of the isolated peptides on H_2_O_2_-induced human umbilical vein endothelial cells (HUVECs).

## Materials and Methods

### Materials

*E. cottonii* was purchased from Taiwan Baijiazhenming Biotechnology Co., Ltd. (Taizhong, China). HUVECs were bought from the Chinese Academy of Sciences (Shanghai, China). Pepsin, alcalase, and flavourzyme were purchased from Shanghai Jingchun Biochemical Technology Co., Ltd. (Shanghai, China). Trypsin and papain were bought from Gibco Life Technologies (Waltham, MA, United States). Methyl thiazolyldiphenyl-tetrazolium bromide (MTT), acetylcysteine (NAc), and DPPH were purchased from Sigma Corporation of America (Ronkonkoma, NY, United States). Sephadex G-25 was purchased from Yuanju Biological Engineering Co., Ltd. (Shanghai, China). SOD, GSH-Px, and MDA assay kits were bought from Nanjing Built Biological Co., Ltd. (Nanjing, China). All other chemicals and reagents were of analytical grade and bought from Sinopharm Chemical Reagent Co., Ltd. (Shanghai, China).

### Extraction of *E. cottonii* Proteins

The powder of *E. cottonii* was soaked in deionized water with a ratio of 1: 40 (g/ml) stirred for 12 h at 26°C and subsequently ultrasonically extracted for 30 min at a frequency of 53 kHz and a power of 100 W using an ultrasonic device of KQ100TDE (Shanghai Precision Instruments Co., Ltd., Shanghai, China). The extracted solution was centrifuged at 7,000 *g* for 15 min, and the supernatant was added into (NH_4_)_2_SO_4_ solution to achieve 10% saturation. After 20 min, the solution was centrifuged at 7,000 *g* for 15 min at 4°C, and the resulted precipitate (referred to ZD10) was collected. The supernatant was added into (NH_4_)_2_SO_4_ solution to achieve 60% saturation and stirred for 20 min. The solution was kept at 4°C for 24 h and freezing centrifuged at 7,000 *g* for 10 min. The resulted precipitate (referred to as ZD60) was collected. The precipitates of ZD10 and ZD60 were separately dissolved in an appropriate amount of deionized water, dialyzed, lyophilized, and reserved at –20°C for further experiment. Protein concentration was determined by the Bradford method (Bradford, 1976).

### Preparation of Antioxidant Peptides From Protein Hydrolysate of *E. cottonii*

**FIGURE 1:**
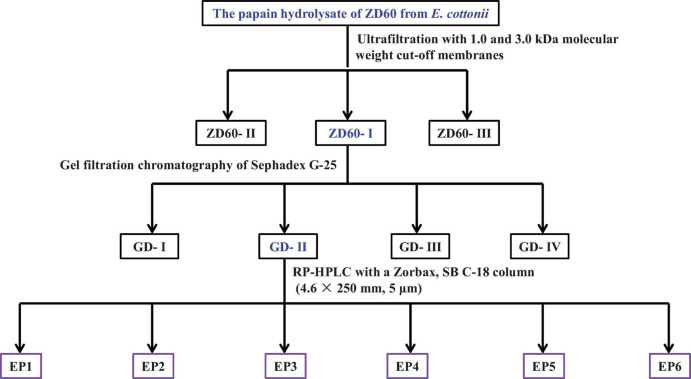
The flow diagram of isolating antioxidant peptides from the papain hydrolysate of ZD60 [proteins precipitated using 60% (NH_4_)_2_SO_4_].

Screening of proteinase species: ZD10 and ZD60 were dissolved (5% w/v) in 0.05 M phosphate-buffered solution (PBS, 0.5 M, pH 7), respectively, and hydrolyzed separately using pepsin (37°C, pH 2), papain (55°C, pH 7), alcalase (50°C, pH 8.5), neutrase (40°C, pH 7.5), and trypsin (40°C, pH 8) with enzyme dose of 1%. After enzymatic hydrolysis of ZD10 and ZD60 for 4 h, the resulted protein hydrolysates were put in boiling water for 15 min and centrifuged at 4,000 *g* for 10 min, and the supernatants were collected to determine their DPPH⋅ scavenging activities (Wang et al., 2013). The papain hydrolysate of ZD60 showed the highest radical scavenging activity, and the flow diagram of antioxidant peptide separation from the hydrolysate is presented in Figure 1.

Ultrafiltration: The papain hydrolysate of ZD60 was fractionated by 1 and 3 kDa molecular weight (MW) cutoff membranes. A number of three fractions termed ZD60-I (MW < 1 kDa), ZD60-II (1–3 kDa), and ZD60-III (> 3 kDa) were prepared and their DPPH⋅ scavenging activities were subsequently measured. Then, ZD60-I with the highest radical scavenging activity was taken for the subsequent experiments.

Gel filtration chromatography: A total of 4 ml of ZD60-I solution (60 mg/ml) was injected into the Sephadex G-25 chromatographic column (2 cm × 120 cm) and eluted using ultrapure water with a flow rate of 0.6 ml/min. The eluent was collected every 2 min and monitored at 280 nm. A number of four subfractions, namely GD-I, GD-II, GD-III, and GD-IV, were collected according to the chromatographic curve at 280 nm. Among them, GD-II showed the highest radical scavenging activity.

Reverse-phase high-performance liquid chromatography (RP-HPLC): The subfraction of GD-II was further separated by Agilent 1200 HPLC System (Agilent Ltd., Santa Clara, CA, United States) on a Zorbax, SB C-18 column (4.6 mm × 25 mm, 5 μm) using a linear gradient of acetonitrile (0–50% in 0–30 min) in 0.05% trifluoroacetic acid at a flow rate of 0.8 ml/min. The elution solution was detected at 214 and 280 nm, and six peptides (EP1–EP6) were collected on their chromatographic peaks and lyophilized.

The identification of amino acid sequences and molecular masses of EP1- EP6: Amino acid sequences and molecular masses of EP1- EP6 were determined according to the previous method described by Chi et al. (2014). In brief, EP1–EP6 were measured through N-terminal amino acid sequencing on an Applied Biosystems 494 protein sequencer (Perkin Elmer/Applied Biosystems Inc., Foster City, CA, United States). The accurate molecular masses of EP1–EP6 were determined using a Q-TOF mass spectrometer (Micromass, Waters, Milford, MA, United States) with an electrospray ionization (ESI) source.

### Evaluation of Radical Scavenging Activities

The radical scavenging assays including DPPH⋅, HO⋅, and O- 2⋅ were carried out according to the previous methods (Supplementary Material 1; Luo et al., 2013; Zhao et al., 2018). The half elimination ratio (EC_50_) was identified as the concentration where a peptide reduced half of the initial radical concentration.

### Cytoprotection of the Isolated Antioxidant Peptides on H_2_O_2_-Induced Oxidative Damaged Model of HUVECs

#### Cell Culture and Viability Assay

The cell culture and viability were carried out according to the previous methods (Cai et al., 2019). In brief, HUVECs were cultured in Dulbecco’s modified eagle medium containing 10% fetal bovine serum (FBS) and 1% penicillin–streptomycin at 5% CO_2_ atmosphere and 37°C for 24 h. Subsequently, HUVECs were further cultured in 200 μm sample solution for 12 h and washed two times with PBS (0.5 M, pH 7). After that, MTT with a final concentration of 0.5 mg/ml was added and incubated for 4 h. Then, the formazan crystals formed by active HUVECs were dissolved in 150 μl of dimethyl sulfoxide. The optical density (OD) value at 570 nm was measured, and the cell viability was calculated based on the following equation:


Cellviability=(OD/sampleOD)control×100%.

#### Establishment of H_2_O_2_-Induced Oxidative Damaged Model of HUVECs

Human umbilical vein endothelial cells were seeded on a 96-well plate at a density of 5 × 10^4^ cells per well and cultured for 24 h. After that, 20 μl of H_2_O_2_ (final concentrations were 0, 100, 200, 300, 400, 500, 600, 700, 800, and 900 μm, respectively) was added and cultured for 2, 6, 12, 24, and 48 h, respectively. After that, the cells were incubated with 20 μl of MTT for 4 h, and the OD values at 570 nm were measured. According to the previous literature, the concentration of H_2_O_2_ induced approximately 50% cell viability was chosen as the condition of oxidative damaged cells (Han et al., 2017).

#### Determination of the Levels of ROS

The ROS levels in different HUVECs groups were measured according to the previous method (Cai et al., 2019). In brief, HUVECs were preincubated with peptide samples at the concentration of 200 μm for 12 h and then incubated with 300 μm H_2_O_2_ for 2 h. After that, the HUVECs were washed with PBS (0.5 M, pH 7) and incubated with 10 μm 2′,7′-dichlorodihydro fluorescein (DCF) diacetate in a fresh culture medium for 0.5 h. ROS levels were indicated by DCF fluorescence and quantified using excitation and emission filters of 485 ± 25 nm and 525 ± 25 nm, respectively, on a BD FACSCalibur flow cytometer (BD Biosciences, San Diego, C, United States). The results were expressed as% of the control value.

#### Effects of EP3-EP6 on SOD, GSH-Px, and MDA in H_2_O_2_-Induced Oxidative Damaged Model of HUVECs

Human umbilical vein endothelial cells were cultured in 6-well plates (1 × 10^6^ cells/well). EP3–EP6 with the final concentration of 200 μm were added into the protection groups, respectively, and the model and protection groups were exposed to H_2_O_2_. Finally, the cell lysis buffer (500 ml) was added to each well on ice. The mixed samples were lysed for 0.5 h and centrifuged for 10 min with 12,000 *g* at 4°C. The resulting liquid supernatants were refrigerated at 4°C. The indicators including SOD, GSH-Px, and MDA were measured within 6 h using assay kits according to the manufacturer’s protocols, and the bicinchoninic acid (BCA) method was applied to determine the protein concentrations for normalizing their levels.

#### Determination of Apoptosis by Annexin-V-PI Staining

The apoptosis assay was performed according to the previous method (Han et al., 2017). About 1.6 ml of HUVECs was inserted into a 6-well plate and cultured for 24 h, and then, 200 μl of the isolated antioxidant peptides with final concentrations of 100 and 200 μm was added into the experimental groups, respectively; the model and blank groups were added with 200 μl of serum-free culture medium (SFM). After being cultured for 3 h, 200 μl of SFM containing H_2_O_2_ with the final concentration of 300 μm was added into the sample and model groups, while 200 μl of SFM fluid without H_2_O_2_ for the blank group. After culturing for 6 h, HUVECs with a density of 1 × 10^6^/ml were resuspended in 300 μl PBS (0.5 M, pH 7). Then, 100 μl of the cell suspension, 5 μl of annexin V-fluorescein isothiocyanate (AV-FITC), and 5 μl of propidine iodide (PI) (0.05 mg/ml) were added into a 1.5-ml centrifuge tube and reacted for 15 min at 25°C. After that, 400 μl of PBS (0.5 M, pH 7) was added, mixed, and placed in a flow cytometer for detection.

### Statistical Analysis

All data were expressed as the mean ± standard deviation (SD, *n* = 3). The acquired data were analyzed by the one-way ANOVA test using the software of SPSS 19 (SPSS Corporation, Chicago, IL, United States). The differences among the parameter means were analyzed using Duncan’s multiple range test, and the level of significance was set as *p* < 0.05, *p* < 0.01, and *p* < 0.001.

## Results and Discussion

### Preparation of Antioxidant Peptides of *E. cottonii*

#### Screening of Proteinase Species

Using single factor experiment, the extraction conditions of crude proteins of *E. cottonii* were optimized and presented in Supplementary Material 2. The data indicated that the yield of crude proteins of *E. cottonii* was 10.77 ± 0.42%, which was higher than the protein content (9.76 ± 1.33%) as reported by Matanjun et al. (2008). The crude proteins of *E. cottonii* were divided into two fractions of ZD10 and ZD60 using 10 and 60% (NH_4_)_2_SO_4_, respectively. The yield of ZD60 was 52.77 ± 1.42 g/100 g crude proteins, which was significantly higher than that of ZD10 (13.40 ± 0.89 g/100 g).

**FIGURE 2:**
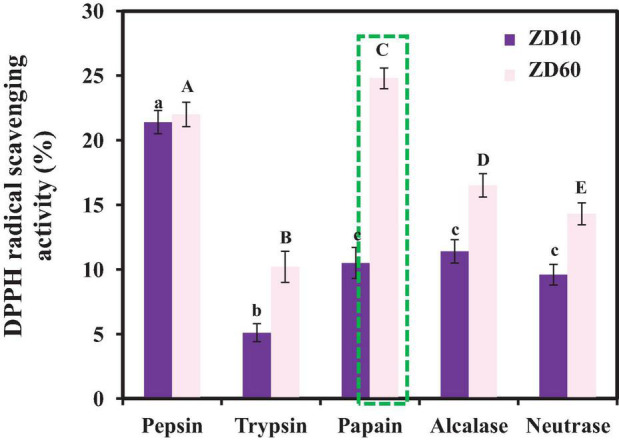
DPPH⋅ scavenging activities of different enzymatic hydrolysates of ZD10 [proteins precipitated using 10% (NH_4_)_2_SO_4_] and ZD60 [proteins precipitated using 60% (NH_4_)_2_SO_4_] at the concentration of 10 mg/ml. All data were presented as the mean ± SD (*n* = 3). ^a– c or A– E^ Values with the different letters indicate a significant difference (*p* < 0.05).

2,2-diphenyl-1-picrylhydrazyl radical H⋅ scavenging activity was used as an evaluation indicator to compare the effects of proteinase species on the antioxidant activity of the enzymatic hydrolysates. Figure 2 revealed that the antioxidant effects of the protein hydrolysates of *E. cottonii* were significantly influenced by the type of protease species at the concentration of 10 mg/ml. DPPH⋅ scavenging rates of hydrolysates from ZD60 were all better than those from ZD10 using the same protease. In addition, the enzymatic hydrolysate of ZD60 prepared by papain showed the highest DPPH⋅ scavenging rate (25.91 ± 0.24%). Taking into account the protein yield and the radical scavenging activity, the enzymatic hydrolysate prepared using papain with a yield of 36.31 ± 2.53 g/100 g ZD60 was selected for subsequent experiments. At present, bioactive peptides are often prepared from animal or plant proteins using solvent extraction, chemical treatment, enzymatic hydrolysis, and microbial fermentation (Alasalvar et al., 2010), and enzymatic hydrolysis is considered to be the higher priority method in food and industrial production due to its rapidity and convenience, no organic solvent residues, and no release of harmful substances (Sila and Bougatef, 2016). In addition, different proteinases may produce hydrolysates from the same protein resources with significantly different biological activities due to their substrate specificity and hydrolysis sites. Our results illustrate that plant-derived papain is the best protease for obtaining peptides with the best DPPH⋅ scavenging activity from *E. cottonii* proteins.

#### Ultrafiltration Separation of ZD60

**FIGURE 3:**
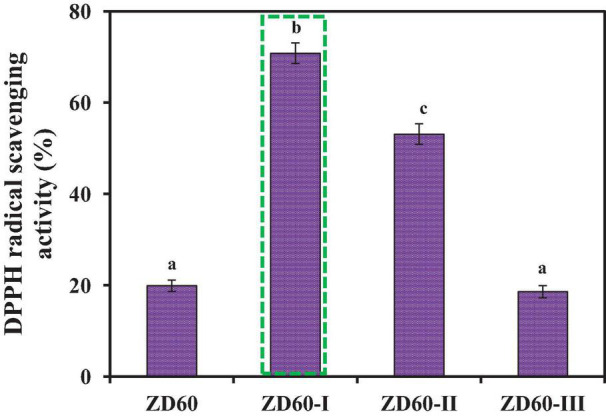
DPPH⋅ scavenging activities of papain hydrolysate of ZD60 and its three subfractions (ZD60-I to ZD60-III) at the concentrations of 10 mg/ml, respectively. All data were presented as the mean ± SD (*n* = 3). ^a–c^ Values with the different letters indicate a significant difference (*p* < 0.05).

The subfractions of ZD60-I (MW < 1 kDa), ZD60-II (1–3 kDa), and ZD60-III (> 3 kDa) were prepared using 1 and 3 kDa MW cutoff membranes. As presented in Figure 3, the DPPH⋅ scavenging rate of ZD60-I was 70.80 ± 1.28% at the concentration of 10 mg/ml, which was significantly higher than those of ZD60 (19.91 ± 1.14%), ZD60-II (53.14 ± 1.35%), and ZD60-III (18.63 ± 1.44%) (*p* < 0.05). These data suggested that the enzymatic peptides of *E. cottonii* with lower MW were more effectively interacted with DPPH⋅ to block the oxidation reaction, which was consistent with the previous reports, and the antioxidant capacities of hydrolysates were negatively correlated with their MW (Li et al., 2013; Sila and Bougatef, 2016; Zhao et al., 2018). Therefore, ZD60-I was selected for subsequent separation.

#### Gel Filtration Chromatography of ZD60-I

**FIGURE 4:**
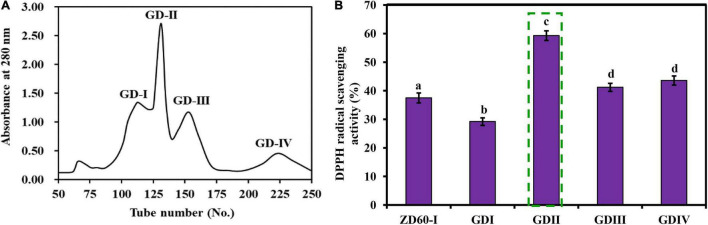
Elution profile of ZD60-I in Sephadex G-25 chromatography **(A)** and the DPPH⋅ scavenging rates of four subfractions (GD-I to GD-IV) from ZD60-I at the concentration of 10 mg/ml, respectively **(B)**. All data were presented as the mean ± SD (*n* = 3). *^a^*– *^d^* Values with the different letters indicate a significant difference (*p* < 0.05).

As shown in Figure 4A, ZD60-I was subjected to a Sephadex G-25 gel chromatography and divided into four subfractions (referred to as GD-I to GD-IV). At the concentration of 10 mg/ml, the DPPH⋅ scavenging rate of GD-II was 59.39 ± 2.09%, which was significantly higher than those of GD-I (30.32 ± 1.89%), GD-III (42.21 ± 1.49%), and GD-IV (45.21 ± 1.84%) (*p* < 0.05) (Figure 4B). Thereby, GD-II was selected for subsequent experiments.

#### Isolation and Determination of the Antioxidant Peptides From GD-II by RP-HPLC

**FIGURE 5:**
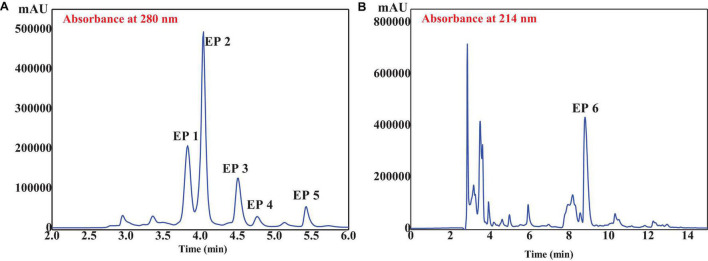
RP-HPLC profile of GD-II on a Zorbax C18 column (4.6 mm × 250 mm, 5 μm) at 280 **(A)** and 214 nm **(B)** wavelength.

GD-II was further separated by RP-HPLC on a Zorbax C18 column using a linear gradient of acetonitrile (0.05% TFA). As shown in Figure 5, six peptides with retention times of 3.81 min (EP1), 4.06 min (EP2), 4.52 min (EP3), 4.76 min (EP4), 5.47 min (EP5), and 8.98 min (EP6) were detected and collected at 280 nm (Figure 5A) and 214 nm (Figure 5B), respectively.

A total of six isolated peptides (EP1–EP6) from the subfraction of GD-II were analyzed by Procise Protein/peptide Sequencer for the N-terminal amino-acid sequence and Q-TOF mass spectrometer for molecular mass, and their amino acid sequences were determined to be Thr-Ala (TA, EP1), Met-Asn (MN, EP2), Tyr-Ser-Lys-Thr (YSKT, EP3), Tyr-Ala-Val-Thr (YAVT, EP4), Tyr-Leu-Leu (YLL, EP5), and Phe-Tyr-Lys-Ala (FYKA, EP6) with MWs of 190.21, 263.33, 497.55, 452.51, 407.51, and 527.62 Da, respectively (Table 1). The determined molecular masses were in agreement with their theoretical masses calculated according to their peptidic sequences (Table 1). In Figure 5B, the peaks with retention time (R*t*) around 3 and 8 min have been collected, but the peptide sequences cannot be identified due to serious overlap. In our further research, we will focus on the purification of these parts and the peaks at 10–13 min and conduct a systematic comparison with the activities of EP1–EP6.

**TABLE 1:** Retention time (R*t*), amino acid (AA) sequences, molecular masses, and radical scavenging activities of six antioxidant peptides (EP1-EP6) from *E. cottonii*.

	R*t* (min)	AA sequence	Theoretical mass/observed mass (Da)	Half elimination ratio (EC_50_, mm)		
				DPPH⋅	HO⋅	O- 2⋅
EP1	3.81	TA	190.20/190.21	8.96 ± 0.37 ^a^	5.97 ± 0.25 ^a^	4.91 ± 0.11 ^a^
EP2	4.06	MN	263.31/263.33	3.57 ± 0.26 ^b^	4.35 ± 0.22 ^b^	3.19 ± 0.26 ^b^
EP3	4.52	YSKT	497.54/497.55	1.71 ± 0.21 ^d^	2.87 ± 0.15 ^c^	1.34 ± 0.11 ^d, e^
EP4	4.76	YAVT	452.50/452.51	1.45 ± 0.20 ^d^	1.24 ± 0.10 ^e^	0.56 ± 0.16 ^f^
EP5	5.47	YLL	407.50/407.51	0.52 ± 0.19 ^e^	2.57 ± 0.20 ^d^	1.61 ± 0.17 ^c, d^
EP6	8.98	FYKA	527.61/527.62	2.56 ± 0.24 ^c^	3.54 ± 0.25 ^c^	1.91 ± 0.11 ^c^
GSH	–	–	–	1.37 ± 0.20 ^d^	0.72 ± 0.04 ^f^	1.11 ± 0.12 ^e^

*The data are presented as the mean ± SD (n = 3).*

*^a–f^Values with different letters in each column indicate significant difference (p < 0.05).*

### Evaluation of Antioxidant Activities

#### Radical Scavenging Activities

The oxidation process in biological systems is very complex, the mechanisms of different types of active ingredients to play their antioxidant effects may be completely different, and there is currently no method that can directly and accurately screen all antioxidant active ingredients. A large number of free radicals will be generated in the process of organism metabolism, and the excessive accumulation of these free radicals will cause the body’s peroxidation reaction, which will lead to various diseases. Therefore, free radical scavenging activities such as DPPH⋅, HO⋅, and O- 2⋅ have been used widely to rapidly screen antioxidant active substances *in vitro*.

The EC_50_ values of six isolated antioxidant peptides (EP1–EP6) on DPPH⋅, HO⋅, and O- 2⋅ are presented in Table 1. The data showed that the EC_50_ value of EP5 on DPPH⋅ (0.52 ± 0.092 mm) was significantly lower than those of other five peptides and the positive control of glutathione (GSH) (EC_50_ 1.37 ± 0.10 mm) (*p* < 0.05), which is also less than those peptides from skipjack tuna (*Katsuwonus pelamis*) roe (AEM: 0.72 ± 0.010; YEA: 0.61 ± 0.031; AEHNH: 0.55 ± 0.018; YVM: 0.70 ± 0.036 mm) (Wang et al., 2022), Antarctic krill (VEKGK: 0.54 ± 0.029; IEKT: 0.91 ± 0.082; IDSQ: 1.02 ± 0.90; IEN: 1.45 ± 0.20 mm) (Wang et al., 2021), skipjack tuna (*K. pelamis*) head (VEE: 1.00 ± 0.43 mm) (Zhang et al., 2019a), and *Euphausia superba* (QYPPMQY: 1.67 ± 0.16; SLPY: 2.47 ± 0.075; EYEA: 2.69 ± 0.54; YMNF: 2.92 ± 0.077; NQM: 4.34 ± 0.17 mm) (Zhang et al., 2021).

The EC_50_ values of EP4 on HO⋅ and O- 2⋅ were 1.24 ± 0.10 mm and 0.56 ± 0.16 mm, respectively, which were significantly lower than those of other five peptides (EP1: 5.97 ± 0.25, 4.91 ± 0.11; EP2: 4.35 ± 0.22, 3.19 ± 0.26; EP3: 2.87 ± 0.15, 1.34 ± 0.11; EP5: 2.57 ± 0.20, 1.61 ± 0.17; EP6: 3.54 ± 0.25,1.91 ± 0.11 mm, respectively) (*p* < 0.05). At the same time, they were also notably less than those peptides from skipjack tuna (*K. pelamis*) roe (AEM: 1.31 ± 0.043, 1.00 ± 0.052; TVM: 2.70 ± 0.19, 3.06 ± 0.18; YEA: 1.25 ± 0.13, 0.80 ± 0.058; VDTR: 3.11 ± 0.17, 3.32 ± 0.25 mm, respectively) (Wang et al., 2022), Antarctic krill (VEK: 4.63 ± 0.11, 0.86 ± 0.061; VEKT: 3.22 ± 0.12, 1.81 ± 0.67; AEKTR: 2.90 ± 0.13, 6.60 ± 0.59; VEKGK: 2.91 ± 0.023, 2.98 ± 0.082, LKPGN: 1.36 ± 0.011, 1.42 ± 0.045 mm, respectively) (Wang et al., 2021), skipjack tuna (*K. pela mis*) head (DAGPYGPI: 2.17 ± 0.076, 1.92 ± 0.014; VEE: 6.47 ± 0.029, 4.77 ± 0.029 mm, respectively) (Zhang et al., 2019a), and *E. superba* (AFLWA: 2.35 ± 0.12, 1.79 ± 0.055; SLPY: 1.73 ± 0.056, 1.65 ± 0.17; EYEA: 1.85 ± 0.022, 1.55 ± 0.11; NVPDM: 3.20 ± 0.056, 5.07 ± 0.27 mm) (Zhang et al., 2021). In addition, the EC_50_ value of EP4 on O- 2⋅ was significantly lower than the positive control of GSH (1.11 ± 0.12 mm, *p* < 0.05). Compared with the positive control GSH, EP3 and EP6 showed moderate scavenging activities on DPPH⋅, HO⋅, and O- 2⋅, whereas EP1 and EP2 had weak activities. In summary, EP4 and EP5 showed outstanding radical scavenging capacities *in vitro*, among which EP5 had the strongest scavenging ability on DPPH⋅, and EP4 had the strongest scavenging ability on HO⋅ and O- 2⋅ among six isolated peptides (EP1–EP6). Therefore, peptides EP3–EP6 were selected for further activity studies.

#### Cytoprotections of EP3–EP6 on H_2_O_2_-Induced Oxidative Damaged Model of HUVECs

##### Protective Abilities of EP3–EP6 on H_2_O_2_-Induced Oxidative Damaged Model of HUVECs

Human umbilical vein endothelial cells are currently considered one of the best target cells in the treatment of cardiovascular diseases and play an important role in a series of physiological and pathological processes such as inflammation, wound repair, angiogenesis, and atherosclerosis. Once the excessive accumulation of free radicals causes the body’s oxidative stress response, which leads to the damage of HUVECs, it will lead to the damage of the vascular barrier function, which will lead to the occurrence of atherosclerosis, hypertension, and other cardiovascular diseases.

To evaluate the cytoprotections of EP3–EP6 on oxidative damaged HUVECs, the oxidative damage model of HUVECs was established using H_2_O_2_ at concentrations of 0–900 μm and administrated for 2–48 h, respectively (Supplementary Material 3). Considering the cells and culturing cycle, the treatment of H_2_O_2_ at a concentration of 300 μm for 6 h was suggested to establish the oxidative damage model of HUVECs. Through verification tests, the cell viability was 47.22 ± 1.64% of the blank group (*p* < 0.001) at the selected conditions.

**FIGURE 6:**
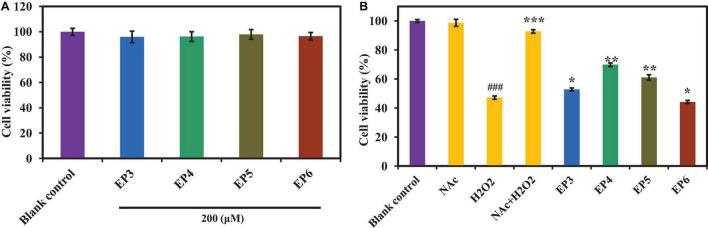
Effects of the antioxidant peptides (EP3–EP6) on HUVEC viabilities **(A)** and their protective effects against oxidative damage in H_2_O_2_-induced HUVEC model **(B)**. Blank control refers to the viability of the normal cells without any treatment; Acetylcysteine (NAc) at the concentration of 100 μm was used as the positive control; H_2_O_2_ refers to H_2_O_2_-induced oxidative damaged model (300 μm for 6 h); NAc + H_2_O_2_ refers to NAc treated with H_2_O_2_-induced HUVECs at the concentration of 100 μm; peptide + H_2_O_2_ refers to EP3–EP6 treated with H_2_O_2_-induced HUVECs at the concentration of 200 μm, respectively. All data were presented as the mean ± SD (*n* = 3). **(A)** Values in each column have no significant difference (*p* > 0.05). **(B)** ^###^*p* < 0.001 vs. the blank control group; ^***^p < 0.001, ^**^p < 0.01, and *p < 0.05 vs. the H_2_O_2_-treated group.

As shown in Figure 6A, the cell viabilities of peptide-treated groups (EP3–EP6) were higher than 95% of the blank control group at the concentration of 200 μm, which indicated that EP3–EP6 have no significant effects on the proliferation of HUVECs. Then, EP3–EP6 (200 μm) were used as the drug-administered groups. Figure 6B indicated that the cell viability of the positive control group (NAC + H_2_O_2_) has risen to 91.32 ± 0.86%, which was extremely significantly superior (*p* < 0.001) to that of the oxidative damage model group (H_2_O_2_) 47.23 ± 1.64%. In addition, in the treated groups of EP4 and EP5, the cell viabilities had risen to 69.92 ± 3.37% and 61.14 ± 2.73%, respectively, which were very significantly higher than that of the model group (47.23 ± 1.64%) (*p* < 0.01). At the same time, the cell viabilities in EP3 and EP6 were 52.81 ± 1.47% and 56.12 ± 1.79%, respectively, which were significantly better than that of the model group (*p* < 0.05) (Figure 6B). Therefore, these results indicated that EP4 and EP5 could significantly protect HUVECs from H_2_O_2_-induced oxidative damage.

##### Effects of EP3–EP6 on the Levels of ROS in H_2_O_2_-Induced Oxidative Damaged Model of HUVECs

**FIGURE 7:**
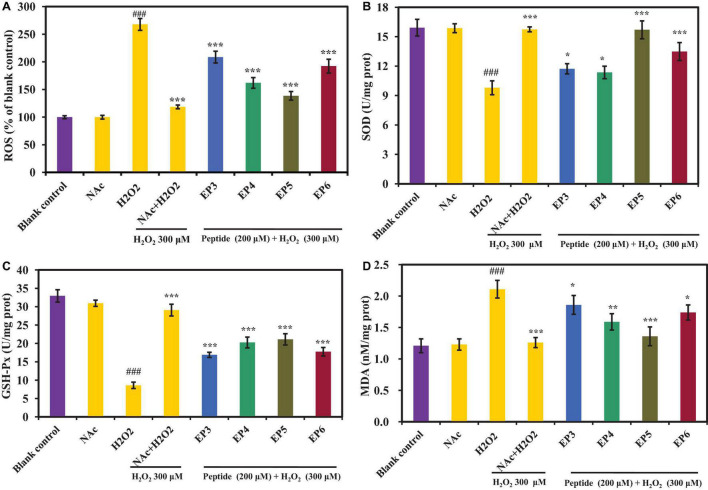
Effects of the antioxidant peptides (EP3–EP6) on the levels of reactive oxygen species (ROS) **(A)**, superoxide dismutase (SOD) **(B)**, glutathione peroxidase (GSH-Px) **(C)**, and malondialdehyde (MDA) **(D)** in oxidative damage HUVECs at the concentration of 200 μm. Blank control refers to the levels of ROS, SOD, GSH-Px, and MDA in normal cells; NAc column refers to NAc on normal cells at the concentration of 100 μm; H_2_O_2_ column refers to H_2_O_2_-induced oxidative damaged model (300 μm for 6 h); NAc + H_2_O_2_ refers to NAc treated with H_2_O_2_-induced HUVEC model cells at the concentration of 100 μm; peptide + H_2_O_2_ refers to EP3–EP6 treated with H_2_O_2_-induced oxidative damaged model cells at the concentration of 200 μm, respectively. The data were presented as the mean ± SD (*n* = 3). ^###^*p* < 0.001 vs. the blank control group; ^***^p < 0.001, ^**^p < 0.001, and *p < 0.05 vs. the H_2_O_2_-treated group.

Figure 7A shows the effects of EP3–EP6 on the ROS levels in H_2_O_2_ oxidative damaged HUVECs. In the H_2_O_2_-induced oxidative damaged model group, the ROS level was 267.92 ± 10.68%, which was significantly more than that of the blank control group (*p* < 0.001). The results illustrated that the oxidative damaged model of HUVECs was successfully established. Obviously, the ROS levels were extremely significantly attenuated by EP3–EP6 pretreatment at the concentration of 200 μm (*p* < 0.001) compared with the model group. Among them, EP4 and EP5 showed better scavenging activities on ROS than the other groups, which induced that the ROS levels increased from 267.92 ± 10.68 to 162.13 ± 9.64 and 138.61 ± 7.81%, respectively. In cells, the ROS are generated as the byproducts of cellular respiration in mitochondria (Homayouni-Tabrizi et al., 2017). Excessive ROS can cause oxidative stress due to their damage to biomolecules including DNA, proteins, and lipids, which was thought of as the pathogenesis of some chronic diseases (Cai et al., 2019). The present data indicated that EP3–EP6, especially EP4 and EP5, could protect HUVECs from oxidative stress by decreasing the ROS levels.

##### Effects of EP3–EP6 on the Levels of SOD, GSH-Px, and MDA in H_2_O_2_-Induced Oxidative Damaged Model of HUVECs

In this part, SOD, GSH-Px, and MDA levels were measured to explain the cytoprotective mechanisms of EP3–EP6 on the H_2_O_2_-induced HUVEC model. As shown in Figures 7B–C, the levels of SOD (10.03 ± 0.42 U/mg prot) and GSH-Px (9.32 ± 0.87 U/mg prot) were extremely significantly decreased in H_2_O_2_-induced HUVECs, compared with normal control (15.92 ± 0.85 U/mg prot and 32.63 ± 0.41 U/mg prot, respectively) (*p* < 0.001). In comparison, the levels of SOD and GSH-Px were significantly increased (to EP3: 11.93 ± 0.35 U/mg prot, 17.23 ± 0.59 U/mg prot; EP4: 11.41 ± 0.39 U/mg prot, 21.04 ± 1.42 U/mg prot; EP5: 15.71 ± 0.91 U/mg prot, 21.13 ± 1.53 U/mg prot; EP6: 13.24 ± 0.88 U/mg prot, 17.91 ± 0.70 U/mg prot, respectively) than those of the H_2_O_2_ damaged group when incubated with 200 μm of EP3–EP6 (*p* < 0.05). Of which, EP5 showed the highest ability to increase the levels of SOD and GSH-Px among EP3–EP6. Moreover, the SOD level in the group incubated with EP5 was 15.71 ± 0.91 U/mg prot, which has no significant difference from that (15.92 ± 0.85 U/mg prot) of the control group (*p* > 0.05). It means that EP5 can increase the ROS to the normal level in oxidatively damaged cells. On the other hand, the recovery abilities of EP4 and EP5 on intracellular GSH-Px to oxidatively damaged cells are relatively consistent, both better than EP3 and EP6. Figure 7D shows that the MDA level of the H_2_O_2_-induced group was 2.11 ± 0.14 nM/mg prot, which was extremely significantly higher than that of the control group (1.21 ± 0.11 nM/mg prot) (*p* < 0.001). Pretreated with EP3–EP6 at the concentration of 200 μm, the MDA levels of peptides groups significantly decreased from 2.11 ± 0.14 nM/mg prot to 1.86 ± 0.15 nM/mg prot (EP3), 1.59 ± 0.13 nM/mg prot (EP4), 1.36 ± 0.15 nM/mg prot (EP5), and 1.74 ± 0.12 nM/mg prot (EP6), respectively (*p* < 0.05). ROS are continuously generated inevitably under normal physiological conditions and efficiently scavenged by endogenous antioxidant defense systems, including SOD and GSH-Px, which can maintain optimal cellular health through deactivating ROS before they attack cellular components (Chi et al., 2015a; Tao et al., 2018; Cai et al., 2019). In addition, MDA usually serves as an indicator to evaluate the degree of oxidative damage because it is one of the key products of membrane lipid peroxidation (Zhao et al., 2016). The previous literature reported that some protein hydrolysates displayed intracellular antioxidant functions through controlling the levels and gene expression of antioxidant enzymes in cells and organisms, such antioxidant oligopeptides FPYLRH, FWKVV, and FMPLH, derived from muscle hydrolysate of miiuy croaker, played their protective functions through increasing the levels of antioxidant enzymes including SOD and GSH-Px and decreasing the levels of ROS and MDA in oxidative-damaged HUVECs (Cai et al., 2019; Wang et al., 2021). GPEGPMGLE, EGPFGPEG, and GFIGPTE, prepared from collagen hydrolysate of redlip croaker (*Pseudosciaena polyactis*) scales, could protect HepG2 cells from H_2_O_2_-induced oxidative damage through activating intracellular antioxidant enzymes of SOD, CAT, and GSH-Px and decreasing the levels of ROS and MDA (Wang et al., 2020b); Zhang et al. reported that IYVVDLR and IYVFVR from the hydrolysate of soybean protein could protect Caco-2 cells from H_2_O_2_-induced oxidative damage, *via* significantly downregulating intracellular ROS generation and lipid peroxidation, upregulating GSH synthesis, enhancing activities of CAT and GSH-Px, and inhibiting ROS-mediated inflammatory responses (Zhang et al., 2019b). All the data presented in Figure 7 proved that EP3–EP6 could increase the endogenous antioxidant defense systems including SOD and GSH-Px to protect HUVECs from oxidative damage caused by H_2_O_2_.

##### Effects of EP3–EP6 on HUVEC Apoptosis

**FIGURE 8:**
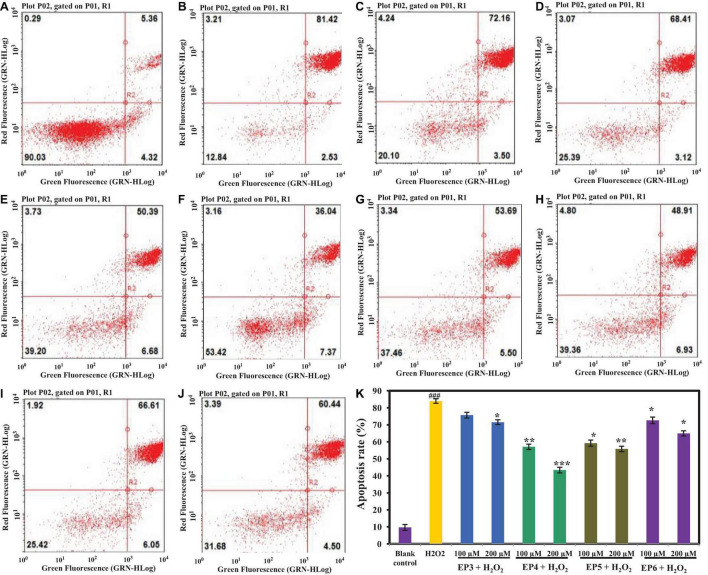
Effects of the antioxidant peptides (EP3–EP6) on HUVEC apoptosis determined by annexin V and propidine iodide (AV-PI) staining. In the Figure 8A–J, the left upper quadrant (LU) represents necrotic cells (annexin-/PI +), the left lower quadrant (LL) represents normal living cells (annexin-/PI-), the right lower quadrant (RL) represents the early apoptotic cells (annexin + /PI-) and the right upper quadrant (RU) represents late apoptotic cells (annexin + /PI +). **(A)** Blank control refers to normal cells without any treatment; **(B)** H_2_O_2_-induced model (300 μm for 6 h); **(C)** EP3 (100 μm) + H_2_O_2_ (300 μm for 6 h); **(D)** EP3 (200 μm) + H_2_O_2_ (300 μm for 6 h); **(E)** EP4 (100 μm) + H_2_O_2_ (300 μm for 6 h); **(F)** EP4 (200 μm) + H_2_O_2_ (300 μm for 6 h); **(G)** EP5 (100 μm) + H_2_O_2_ (300 μm for 6 h); **(H)** EP5 (200 μm) + H_2_O_2_ (300 μm for 6 h); **(I)** EP6 (100 μm) + H_2_O_2_ (300 μm for 6 h); **(J)** EP6 (200 μm) + H_2_O_2_ (300 μm for 6 h); **(K)** the apoptosis rates of HUVECs when the concentrations of blank control, H_2_O_2_-induced model group, 100 μm and 200 μm EP3–EP6 + H_2_O_2_ (300 μm for 6 h) (^###^*p* < 0.001 vs. blank control group; ^***^p < 0.001, ^**^p < 0.01, *p < 0.05 vs. H_2_O_2_-induced model group).

As shown in Figure 8 and Supplementary Table 2, the effects of EP3–EP6 on apoptosis of HUVECs were analyzed by annexin V and PI double-staining methods, and four cell subpopulations were distinguished and quantified by flow cytometry (Jia et al., 2018). The apoptosis rate of the H_2_O_2_-induced model group was 83.95 ± 1.67% (Figure 8B), which was significantly higher than that of the blank control group (9.68 ± 1.21%) (Figure 8A) (*p* < 0.001). Figures 8C–J and Supplementary Table 2 showed that EP3–EP6 could reduce the apoptosis rate of H_2_O_2_-induced HUVECs at different levels. EP4 and EP5 (200 μm) could significantly reduce the rate of apoptosis to 43.41 ± 1.08% (*p* < 0.01) and 55.84 ± 1.45% (*p* < 0.001), respectively (Figure 8K).

Figures 8A,B and Supplementary Table 2 show that the early apoptosis rates of HUVECs in the blank control group and H_2_O_2_-induced model group were 4.32 ± 0.24 and 2.53 ± 0.19%, respectively, which indicated that H_2_O_2_ could promote the progression of early apoptotic cells to late apoptosis. At the concentrations of 100 and 200 μm, the early apoptosis rates of HUVECs in EP4 (6.68 ± 0.28% and 7.37 ± 0.31%) and EP5 (5.50 ± 0.29% and 6.93 ± 0.32%) groups were 2–3 times higher than that (2.53 ± 0.19%) of HUVECs in the model group (Figures 8E–H and Supplementary Table 2) (*p* < 0.05). Apoptosis is a fundamental biological phenomenon of cells and plays an essential role in the removal of unwanted or abnormal cells in multicellular organisms, the evolution of organisms, maintaining the stability of the internal environment, and the development of multiple systems. Apoptosis is not only a special type of cell death but also has important biological significance and complex molecular biological mechanisms. The process of apoptosis cannot be reversed, but it can be delayed. The results above clearly indicated that EP4 and EP5 could effectively delay H_2_O_2_-induced apoptosis by preventing the progression of cells from early apoptosis to late apoptosis.

## Discussion

At present, many bioactive peptides with excellent antioxidant capacities have been screened and identified from different hydrolysates of marine protein resources, such as LNGDVW derived from protein hydrolysate of *C. ellipsoidea* (Ko et al., 2012), EDIVCW, MEPVW, and YWDAW from Monkfish (*L. litulon*) muscle (Chi et al., 2014), SY identified from the gonad of *Rhopilema esculentum* (Zhang et al., 2018), WEGPK, GPP, and GVPLT prepared from head and skin of *N. septentrionalis* (Chi et al., 2015b,c), and LKPNM and LKP originating from fish protein (Iwaniak et al., 2014). Generally, the antioxidant functions of bioactive peptides are mainly affected by molecular structural properties, such as molecular size, amino acid hydrophobicity, and peptide sequences (Sila and Bougatef, 2016; He et al., 2019; Mirzaei et al., 2020).

Molecular weight is one of the crucial elements impacting the biological and physiological functional characteristics of peptides (Luo et al., 2013; Sila and Bougatef, 2016). Feng et al. (2018) and Zhao et al. (2018) reported that shorter peptides with amino acid residues ranging from 2 to 10 usually displayed higher abilities on inhibition of lipid peroxidation and ROS scavenging than their parent native proteins. A number of six isolated peptides (EP1–EP6) were dipeptides to tetrapeptides and their MWs ranged from 190.20 to 527.61 Da, which was in agreement with those previous reports that peptides with smaller molecular sizes were more convenient to combine with target molecules (Sila and Bougatef, 2016; Zhao et al., 2018; He et al., 2019). Amino acid composition and position play vital roles in the functions of peptides (Zou et al., 2016; Chai et al., 2017). Hydrophobic amino acid residues, such as Pro, Met, Leu, Tyr, Phe, and Val, can improve the accessibility of antioxidant peptides to hydrophobic radicals by enhancing the density of water–lipid interfaces (Farvin et al., 2016; Feng et al., 2018). The aromatic groups of aromatic amino acid residues (Trp, Phe, and Tyr) can directly transfer electrons or protons to stabilize the active oxygen of radicals (Chi et al., 2015c; Sila and Bougatef, 2016). In addition, polar amino acid residues, such as Lys, Glu, and Asp, can be conducive to the activities of metal-chelating and HO⋅ scavenging (Sila and Bougatef, 2016; Zhao et al., 2018). Therefore, Tyr and Lys residues in the sequence of EP3, Tyr, Ala, and Val residues in the sequence of EP4, Tyr, and two Leu residues in the sequence of EP5, and Phe, Tyr, Lys, and Ala residues in the sequence of EP6 were important for their radical scavenging activities and cytoprotection.

The results in Figures 6, 8 indicated that EP4 and EP5 showed better inhibitory effects on H_2_O_2_-induced oxidative damaged HUVECs. On the analysis of structure–activity relationship of EP4 and EP5, their antioxidant activities are majorly dependent on the composition of amino acid sequence and the length of the peptides. Small molecular size helps them more efficient and easier accessibility to the oxidant or antioxidant system, and hydrophobic amino acids increase their solubility at the water–lipid interface and thereby facilitate better interaction with ROS. Finally, antioxidant amino acids change free radicals into a more stable structure or system to inhibit the propagation of the radical-mediated peroxidizing chain reaction by serving as a hydrogen donor, proton donor, and lipid peroxyl radical trap. In addition, EP5 showed a high protective effect on HUVECs against H_2_O_2_-induced oxidative damage by increasing the levels of the antioxidant enzyme system (SOD and GSH-Px) and reducing the level of ROS and MDA. However, EP4 showed a higher ability to decrease the apoptosis rate of H_2_O_2_ oxidative damaged HUVECs than EP5 did. The previous reports indicated that the antioxidant-related parameters were regulated by the Keap1-Nrf2 signaling pathway *in vivo* (Li et al., 2019; Zhang et al., 2020). In addition, Wang et al. (2020a) reported that resveratrol could inhibit oxidative stress-mediated podocyte apoptosis by activating the AMPK signaling pathway in diabetic nephropathy. Choi found that schisandrin A could block C2C12 cells apoptosis induced by H_2_O_2_ through inhibiting poly (ADP-ribose) polymerase degradation by the inactivation of caspase-3 (Choi, 2018). Therefore, the intracellular antioxidant mechanism of EP4 and EP5 is through the activation of the Keap1-Nrf2 pathway or other mechanisms of action, such as activation of the NF-κB, TNF-α, and MAPK pathways, which will be the focus of our future research.

## Conclusion

Papain hydrolysate of ZD60 with high DPPH⋅ scavenging activity was prepared from *E. cottonii*, from which six antioxidant peptides (EP1–EP6) were purified using series separation methods, such as ultrafiltration and chromatography. Their amino acid sequences were determined as Thr-Ala (EP1), Met-Asn (EP2), Tyr-Ser-Lys-Thr (EP3), Tyr-Ala-Val-Thr (EP4), Tyr-Leu-Leu (EP5), and Phe-Tyr-Lys-Ala (EP6). Among them, EP4 and EP5 showed the best radical scavenging activity and good cytoprotection on H_2_O_2_-induced HUVECs through increasing the levels of antioxidant enzyme system including SOD and GSH-Px to decrease the contents of ROS and MDA. Moreover, EP4 and EP5 could effectively inhibit H_2_O_2_-induced apoptosis by preventing the progression of cells from early apoptosis to late apoptosis. The current results indicated that proteolytic antioxidant peptides of *E. cottonii* should have potential applications in pharmaceutical industries. Our future research will focus on the molecular mechanisms of EP4 and EP5 on cell apoptosis and activation of the Keap1-Nrf2 signaling pathway for elucidating their differences in antioxidant activity.

## Data Availability Statement

The datasets presented in this study can be found in online repositories. The names of the repository/repositories and accession number(s) can be found in the article/Supplementary Material.

## Author Contributions

K-LS prepared the manuscript. MG revised the manuscript, responded to the reviewers’ comments, and checked and revised the final version to be published. Y-ZW and X-RL performed the experiment. PW helped to deal with the sample for the experiment and provided the experimental environment and opinions for the experiment. BW conducted the experimental design and revised the manuscript carefully. All authors contributed to the article and approved the submitted version.

## Conflict of Interest

The authors declare that the research was conducted in the absence of any commercial or financial relationships that could be construed as a potential conflict of interest.

## Publisher’s Note

All claims expressed in this article are solely those of the authors and do not necessarily represent those of their affiliated organizations, or those of the publisher, the editors and the reviewers. Any product that may be evaluated in this article, or claim that may be made by its manufacturer, is not guaranteed or endorsed by the publisher.
