# Supplementary material for: Antioxidant Peptides From Protein Hydrolysate of Marine Red Algae Eucheuma cottonii: Preparation, Identification, and Cytoprotective Mechanisms on H2O2 Oxidative Damaged HUVECs
Source: Front Microbiol. 2022 Apr 21;13:791248. doi: 10.3389/fmicb.2022.791248 (PMC9069057; doi:10.3389/fmicb.2022.791248)
Supplement: Supplementary file 1 [file Data_Sheet_1.DOC]

***Supplementary Material***

Antioxidant peptides from protein hydrolysate of marine red algae Eucheuma cottonii: preparation, identification and cytoprotective mechanism on H_2_O_2_ oxidative damaged HUVECs

Kun-Lai Sun ^1,^ *, Yue-Zhen Wang ^1^, Xue-Rong Li ^1^, Peng Wang ^2^, Bin Wang ^1,^ *

^1^ Zhejiang Provincial Engineering Technology Research Center of Marine Biomedical Products, School of Food and Pharmacy, Zhejiang Ocean University, Zhoushan 316022, China

^2^ College of Food Science and Engineering, Ocean University of China, Qingdao 266003, China

* Correspondence: sunqinlai@126.com (K.L.S.); wangbin4159@hotmail.com (B.W.); Tel./Fax: +86-580-255-4781 (B.W.)

**Supplementary material 1**

**1.1. DPPH· scavenging activity**

2.0 mL of sample consisting of distilled water and different concentrations of the analytes were placed in cuvettes, and 500 μL of ethanolic solution of DPPH (0.02%) and 1.0 mL of ethanol were added. A control sample containing the DPPH solution without the sample was also prepared. In the blank, the DPPH solution was substituted with ethanol. The DPPH· scavenging activity was calculated using the following formula:

DPPH· scavenging activity (%) = (A_c_ + A_b_− A_s_)/A_c_ × 100%,

Where A_s_ is the absorbance rate of the sample, A_c_ is the control group absorbance, and A_b_ is the blank absorbance.

**1.2. HO· scavenging activity**

First, 1.0 mL of 1.86 mM 1,10-phenanthroline solution and 2.0 mL of the sample were added to a screw-capped tube and mixed. Then, 1.0 ml of FeSO_4_·7 H_2_O solution (1.86 mM) was added to the mixture. The reaction was initiated by adding 1.0 mL of H_2_O_2_ (0.03%, v/v). After incubating at 37°C for 60 min in water bath, the absorbance of the reaction mixture was measured at 536 nm against a reagent blank. The reaction mixture without any antioxidant was used as the negative control, and a mixture without H_2_O_2_ was used as the blank. The HO· scavenging activity was calculated using the following formula:

HO· scavenging activity (%) = [(A_s_ − A_n_)/(A_b_ − A_n_)] × 100%,

Where A_s_, A_n_, and A_b_ are the absorbance values determined at 536 nm of the sample, the negative control, and the blank after the reaction, respectively.

**1.3. O- 2· scavenging activity**

In the experiment, superoxide anions were generated in 1.0 mL of nitrotetrazolium blue chloride (NBT) (2.5 mM), 1.0 mL of NADH (624.1 mM), and 1 mL of different sample concentrations. The reaction was initiated by adding 1.0 mL of phenazine methosulphate (PMS) solution (120 μM) to the reaction mixture. The absorbance was measured at 560 nm against the corresponding blank after 5-min incubation at 25 °C. The O- 2· scavenging activity was calculated using the following equation:

O- 2· scavenging activity (%) = [(A_c_ − A_s_)/A_c_] × 100%

Where A_c_ is the absorbance without sample and A_s_ is the absorbance with sample.

**Supplementary material 2**

**2.1. Method**

**2.1.1. Preparation of algal protein from** ***E. cottonii***

In order to improve the protein extraction rate, the extraction conditions were optimized by single factor test. After soaking for 12 h, *E. cottonii* powder was intermittently ultrasonically extracted for 30 min at a frequency of 53 kHz, a power of 100%, and a temperature of 26°C (except for the optimized ultrasound time factor). Optimization factors of extraction solvent (deionized water, phosphate buffer solution (pH 7.0), 2% NaCl solution), ratio of material to liquid (1:20, 1:40, 1:60, 1:80, 1:100), and ultrasound time (20, 30, 40, 50, 60 min) were used to analyze the effects of different conditions on the extraction rate of *E. cottonii* algal protein.

Protein concentration was determined using BCA protein Assay Kit and *E. cottonii* protein extraction rate calculation formula:

$$protein extraction rate (\%)=\frac{protein concentration\times0.02\times dilution times}{total mass of E. cottonii}$$

**2.2. Results and Discussion**

**2.2.1. Optimal extraction conditions of *E. cottonii* protein**


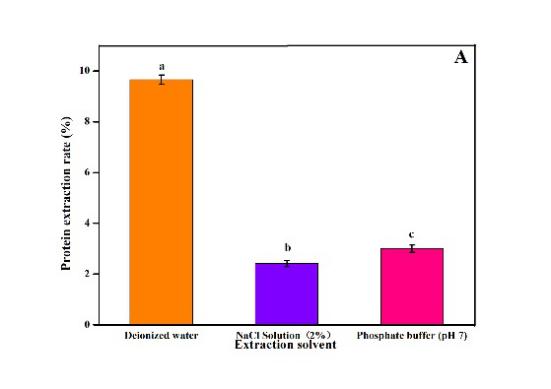

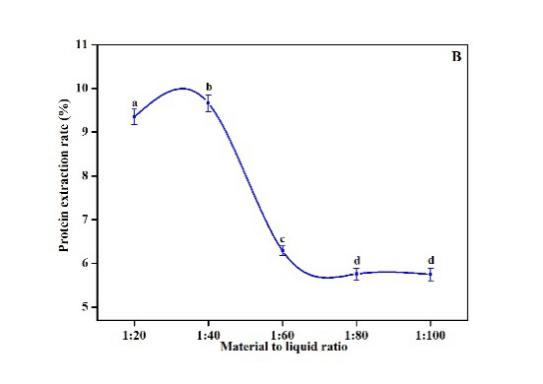

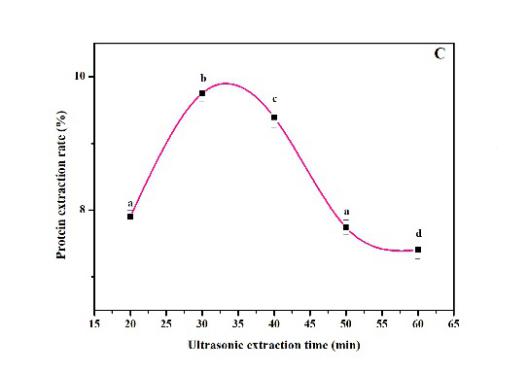


**Fig. S1** The influence of different solvent (A), material to liquid ratio (B) and ultrasonic extraction time (C) to the extraction rate. ^a-d^ Values with different letters indicate significant difference (*P* < 0.05).

Single factor experiments were used to examine the effects of different extraction solvents, ratios of material to liquid, and extraction times on protein extraction rates. The results showed: with deionized water as extraction solvent, the protein extraction rate (9.65 ± 0.38%) was significantly higher than those of phosphate buffer (pH 7.0) and NaCl solution (2.0%) (3.01 ± 0.28%, 2.41 ± 0.24%, respectively) (Fig. S1A). With the increase of the ratio of material to liquid, the protein extraction rate showed an increasing trend at first, then fall to flat. When the ratio of material to liquid was 1: 40, the protein extraction rate was the highest, reaching 9.66 ± 0.19 % (Fig. S1B). With the prolongation of ultrasonic time, the protein extraction rate increased first and then decreased. When the ultrasonic time was 30 min, the protein extraction rate was the highest, reaching 9.75 ± 0.40% (Fig. S1C). Therefore, the optimal extraction conditions for *E. cottonii* protein were: the powder of *E. cottonii* were soaked in deionized water (DW) with a ratio of 1: 40 (g/mL) and stand at 26°C for 12 h, and subsequently ultrasonically extracted for 30 min at a frequency of 53 kHz and at power of 100 W using ultrasonic device of KQ100TDE (Shanghai Precision Instruments co., Ltd., Shanghai, China), and the protein extraction rate was 10.77 ± 0.42%.

**Supplementary material 3**

**3.1. Method**

**3.1.1. Establishment of** **H_2_O_2_-Induced HUVEC Model**

In brief, HUVECs were seeded on a 96-well plate and cultured for 24 h. After that, 20 μL of H_2_O_2_ (final concentrations were 0, 100, 200, 300, 400, 500, 600, 700, 800, and 900 μM, respectively) was added and cultured for 2, 6, 12, 24, and 48 h, respectively. After that, the cells were incubated with 20 μL of MTT for 4 h and the OD value at 570 nm was measured for calculating the cell viability. According to the previous report, the concentration of H_2_O_2_ induced approximate 50% cell survival rate was chosen as the model condition.

**3.2. Results and Discussion**

**3.2.1.** **Establishment of** **Oxidative Damage Model of HUVECs**

In order to evaluate the cytoprotection of EP3-EP6 on oxidative damaged HUVECs, the oxidative damage model of HUVECs was established using H_2_O_2_ at concentrations of 0 ~ 900 μM and administrated for 2-48 h, respectively. Fig. S2 indicated that the cell viability decreased gradually with the increase of H_2_O_2_ concentration and treatment time. Furthermore, the cell viability was close to 50% when HUVECs were treated at the H_2_O_2_ concentration of 300 μM for 2 h, 6 h, 24 h, and 48 h, respectively. In addition, the data in Fig. S2 clearly suggested that the concentrations had significant influences on HUVEC cell viability, while the treatment time showed little effect on the cell viability. Considering the cell and culturing cycle, H_2_O_2_ concentration of 300 μM and treatment for 6 h was suggested to establish the oxidative damage model of HUVECs. Through verification tests, and the HUVEC viability was 47.22 ± 1.64% of the blank group under the conditions of 300 μM for 6 h. The data confirmed that the oxidative damage model of HUVECs induced by H_2_O_2_ could be used in the subsequent experiments.





**Fig. S2** Viability of HUVECs treated with H_2_O_2_ in different concentrations and treatment time. All data are presented as the mean ± SD (n = 3).

**Supplementary material 4**

**Table S1** The raw data of Effects of the antioxidant peptides (EP3-EP6) on HUVEC viability (Figure 6A)

| Groups | mean | SD (n=3) |
| --- | --- | --- |
| Blank control | 100 | 0.845 |
| EP3 | 95.90 | 1.234 |
| EP4 | 96.23 | 1.021 |
| EP5 | 97.88 | 0.998 |
| EP6 | 96.53 | 0.812 |

**Supplementary material 5**

**Table S2** The apoptosis rates of HUVECs in different stages under different conditions.

|  | **Normal living cell (%)** | **Early apoptotic cell (%)** | **Late apoptotic cell (%)** | **Necrotic cell (%)** |
| --- | --- | --- | --- | --- |
| Blank control | 90.03 ± 3.58 ^a^ | 4.32 ± 0.24 ^a^ | 5.36 ± 0.22 ^a^ | 0.29 ± 0.04 ^a^ |
| H_2_O_2_-induced model | 12.84 ± 0.94 ^b^ | 2.53 ± 0.19 ^b^ | 81.42 ± 2.67 ^b^ | 3.21 ± 0.21 ^b,g^ |
| EP3 (100 μM) + H_2_O_2_ | 20.10 ± 1.35 ^c^ | 3.50 ± 0.17 ^c^ | 72.16 ± 3.24 ^b^ | 4.24 ± 0.18 ^c^ |
| EP3 (200 μM) + H_2_O_2_ | 25.39 ± 1.72 ^d^ | 3.12 ± 0.11 ^c^ | 68.41 ± 2.31 ^b^ | 3.07 ± 0.20 ^b^ |
| EP4 (100 μM) + H_2_O_2_ | 39.20 ± 2.27 ^e^ | 6.68 ± 0.28 ^d^ | 50.39 ± 1.98 ^c,d^ | 3.73 ± 0.12 ^d^ |
| EP4 (200 μM) + H_2_O_2_ | 53.42 ± 2.96 ^f^ | 7.37 ± 0.31 ^e^ | 36.04 ± 1.24 ^d^ | 3.16 ± 0.15 ^b,g^ |
| EP5 (100 μM) + H_2_O_2_ | 37.46 ± 1.21 ^e^ | 5.50 ± 0.29 ^f^ | 53.69 ± 3.24 ^c,d^ | 3.34 ± 0.13 ^b,g^ |
| EP5 (200 μM) + H_2_O_2_ | 39.36 ± 1.56 ^e^ | 6.93 ± 0.32 ^d^ | 48.91 ± 1.56 ^c,d^ | 4.80 ± 0.21 ^e^ |
| EP6 (100 μM) + H_2_O_2_ | 25.42 ± 0.97 ^d^ | 6.05 ± 0.27 ^g^ | 66.61 ± 2.24 ^b,c^ | 1.92 ± 0.09 ^f^ |
| EP6 (200 μM) + H_2_O_2_ | 31.68 ± 2.03 ^g^ | 4.50 ± 0.19 ^a^ | 60.44 ± 3.27 ^c,d^ | 3.39 ± 0.14 ^g^ |

The data are presented as the mean ± SD (n=3). ^a–g^ Values with different letters in each column indicate significant difference (*P* < 0.05).
